# Evaluation of atrial septal defects with 4D flow MRI—multilevel and inter-reader reproducibility for quantification of shunt severity

**DOI:** 10.1007/s10334-018-0702-z

**Published:** 2018-08-31

**Authors:** Raluca G. Chelu, Michael Horowitz, Dominica Sucha, Isabella Kardys, Delphine Ingremeau, Shreyas Vasanawala, Koen Nieman, Jean-Francois Paul, Albert Hsiao

**Affiliations:** 1 Department of Radiology, Erasmus MC, ‘s Gravendijkwal 230, Ca207a, 3015 CE Rotterdam, The Netherlands; 2 Department of Cardiology, Erasmus MC, Rotterdam, The Netherlands; 3 Department of Radiology, UCSD, San Diego, CA USA; 4 Department of Radiology, UMC, Utrecht, The Netherlands; 5 Department of Epidemiology, Erasmus MC, Rotterdam, The Netherlands; 6 Department of Radiology, Institut Mutualiste Montsouris, Paris, France; 7 Department of Radiology, Stanford Medical Center, Palo Alto, CA USA; 8 Department of Cardiology, Stanford Medical Center, Palo Alto, CA USA

**Keywords:** 4D flow MRI, Atrial septal defect, Multiple measurements

## Abstract

**Purpose:**

With the hypothesis that 4D flow can be used in evaluation of cardiac shunts, we seek to evaluate the multilevel and interreader reproducibility of measurements of the blood flow, shunt fraction and shunt volume in patients with atrial septum defect (ASD) in practice at multiple clinical sites.

**Materials and methods:**

Four-dimensional flow MRI examinations were performed at four institutions across Europe and the US. Twenty-nine patients (mean age, 43 years; 11 male) were included in the study. Flow measurements were performed at three levels (valve, main artery and periphery) in both the pulmonary and systemic circulation by two independent readers and compared against stroke volumes from 4D flow anatomic data. Further, the shunt ratio (*Q*_p_/*Q*_s_) was calculated. Additionally, shunt volume was quantified at the atrial level by tracking the atrial septum.

**Results:**

Measurements of the pulmonary blood flow at multiple levels correlate well whether measuring at the valve, main pulmonary artery or branch pulmonary arteries (*r* = 0.885–0.886). Measurements of the systemic blood flow show excellent correlation, whether measuring at the valve, ascending aorta or sum of flow from the superior vena cava (SVC) and descending aorta (*r* = 0.974–0.991). Intraclass agreement between the two observers for the flow measurements varies between 0.96 and 0.99. Compared with stroke volume, pulmonic flow is underestimated with 0.26 l/min at the main pulmonary artery level, and systemic flow is overestimated with 0.16 l/min at the ascending aorta level. Direct measurements of ASD flow are feasible in 20 of 29 (69%) patients.

**Conclusion:**

Blood flow and shunt quantification measured at multiple levels and performed by different readers are reproducible and consistent with 4D flow MRI.

**Electronic supplementary material:**

The online version of this article (10.1007/s10334-018-0702-z) contains supplementary material, which is available to authorized users.

## Introduction

Atrial septal defects (ASD) are one of the most common congenital heart defects with an estimated prevalence of 1.6 per 1000 live births [1]. Most prevalent are ostium secundum defects followed by ostium primum and sinus venosus defects [2]. Partial anomalous pulmonary venous connections (PAPVRs) are often associated with ASD, especially with sinus venosus defects. When repaired at a young age, patients with ASD have a life expectancy similar to the general population [3, 4]. Left untreated, patients with large ASD gradually develop pulmonary hypertension, reversal of the left-to-right shunt and eventually right heart failure. In clinical practice, ASD closure is considered for patients with a shunt fraction greater than 1.5 [2].

Multiple imaging modalities are used to detect and delineate these anatomic defects. Transthoracic echocardiography (TTE) is used as a primary screening modality. However, associated pathologies such as PAPVR are more difficult to identify with TTE. When TTE is inconclusive, transesophageal echocardiography (TEE) may be helpful [5]. Alternatively, computed tomography (CT) and magnetic resonance imaging (MRI) are increasingly used.

MRI has shown its incremental value in congenital heart disease (CHD) [6] and ASD in particular [7]. MRI is the gold standard for noninvasive quantification of right heart function and shunt fraction [8, 9]. It may detect intracardiac shunting and additional findings including PAPVR [10]. However, it is performed with numerous breath-holds and relatively long examination times, which may be challenging for cardiac patients.

A promising and rapidly evolving MRI technique is 4D flow imaging, a volumetric, free-breathing acquisition technique of flow velocity data with simultaneous assessment of anatomic structures [11]. The 4D flow MRI allows for flow quantification at any level within the acquired field of view and calculation of cardiac volumes and biventricular function [12–14].

A few studies have evaluated the use of 4D flow MRI for visualization and quantification of cardiac shunts [15–17]. It is not yet clear, however, whether this technique is robust across the range of imaging parameters that might be used in the clinical environment because of differences in body habitus or equipment. In the clinical setting, there may be heterogeneity in imaging techniques because of local preferences or needs for imaging parameters such as the signal-to-noise ratio (SNR), spatial resolution, scanning time, velocity-encoding speed (_Venc_), available equipment, field strengths (1.5 T, 3 T) and patient body habitus. A previous paper showed that it is possible to measure venous flow even when using high _Venc_ [18]. However, uncertainty remains about whether this technique is applicable outside of the research setting. As this technology has recently become more broadly clinically available, we seek to determine in this study whether 4D flow can robustly be used for the evaluation of cardiac shunts at different levels of the vascular tree using 4D flow data acquired across multiple centers—specifically measurements of the blood flow, shunt fraction and shunt volume.

## Methods

### Study design

Cardiac MRI examinations including 4D flow were gathered from four academic centers in the US and Europe in patients referred for evaluation of ASD between December 2014 and January 2017. In three centers, 4D flow was performed as part of the clinical protocol and retrospectively included in this study. Informed consent was waived by the local IRB. In the remaining center, patients were prospectively enrolled and signed informed consent for an MRI including 4D flow. The study protocol was compliant with Declaration of Helsinki and received approval from each local medical ethics committee.

### 4D flow acquisition

At each center, 4D flow MRI acquisition protocols were optimized based on locally available equipment, medications, and clinical requirements. The retrospectively gated 4D flow acquisition was performed using clinical MRI scanners (69% at 1.5 T, 31% at 3 T) (GE Healthcare, Milwaukee, WI, USA) after administration of a gadolinium-based contrast agent. Scan time ranged between 7.46 and 14.75 min (median 10.75 min). All imaging parameters are presented in Table 1.

**Table 1:** Scanning details

	Center 1 (*n* = 4)	Center 2 (*n* = 9)	Center 3 (*n* = 8)	Center 4 (*n* = 8)	Total (*n* = 29)
Field strength (*T*)	1.5 T (100%)	3 T (100%)	1.5 T (100%)	1.5 T (100%)	1.5 T (69%) 3 T (31%)
Contrast agent	Gadobutrol (100%)	Gadofosveset trisodium (67%), gadobenate dimeglumine (33%)	Gadoterate meglumine (50%), gadobenate dimeglumine (50%)	Gadofosveset trisodium (37%), gadobenate dimeglumine (63%)	Gadofosveset trisodium (14%), gadoterate meglumine (31%), gadobutrol (14%), gadobenate dimeglumine (41%)
Resolution acquired (mm)	(1.8– 2.0) × (2.1–2.4) × 2.8	(1.3–2.0) × (1.4–2.4) × (2.4–3.2)	(1.4–1.8) × (2.3–2.8) × 2.6	(1.0–1.8) × (1.4–2.2) × 3.0	(1.3–2.0) × (1.4–2.8) × (2.4–3.2)
Resolution reconstructed (mm)	(1.8–2.0) × (2.1–2.4) × 1.4	(1.3–2.0) × (1.3–2.2) × (1.2–1.6)	(1.4–1.8) × (2.3–2.8) × 1.3	(1.0–1.4) × (1.4–1.8) × 1.5	(1.3–2.0) × (1.3–2.2) × (1.2–1.6)
Temporal resolution (ms)^a^	61.4–62.4	61.2–81.8^**+**^	51.6–76.3	49.3–83.2	49.3–83.2
Heart rate	64–72	66–80	55–103	48–77	48–103
Sinus rhythm	100%	100%	100%	75%	93%
Venc (cm/s)	180–200	150–250	200–500	250	150–500
Scanning time (min)	10.42–11.58	10.75–14.75	7.46–14.75	7.46–10.95	7.46–14.75

Values are ranges (minimum–maximum)

*T* Tesla, *HR* heart rate, *venc* velocity encoding value

^a^In four patients, data were not available

### Post-processing

Data were analyzed using dedicated post-processing software (Arterys Inc, San Francisco, CA). Semiautomatic eddy-current correction was applied [19]. Data were visualized, interpreted for the presence of ASD and classified according to type of septal defect. To evaluate consistency of the data across the vascular tree, shunt quantification was performed at multiple levels, as described below. Further, to evaluate intraobserver reproducibility, background correction and measurements were done by two readers independently (with 4 and 1 year of experience with 4D flow).

#### ASD visualization and classification

**Fig. 1:**
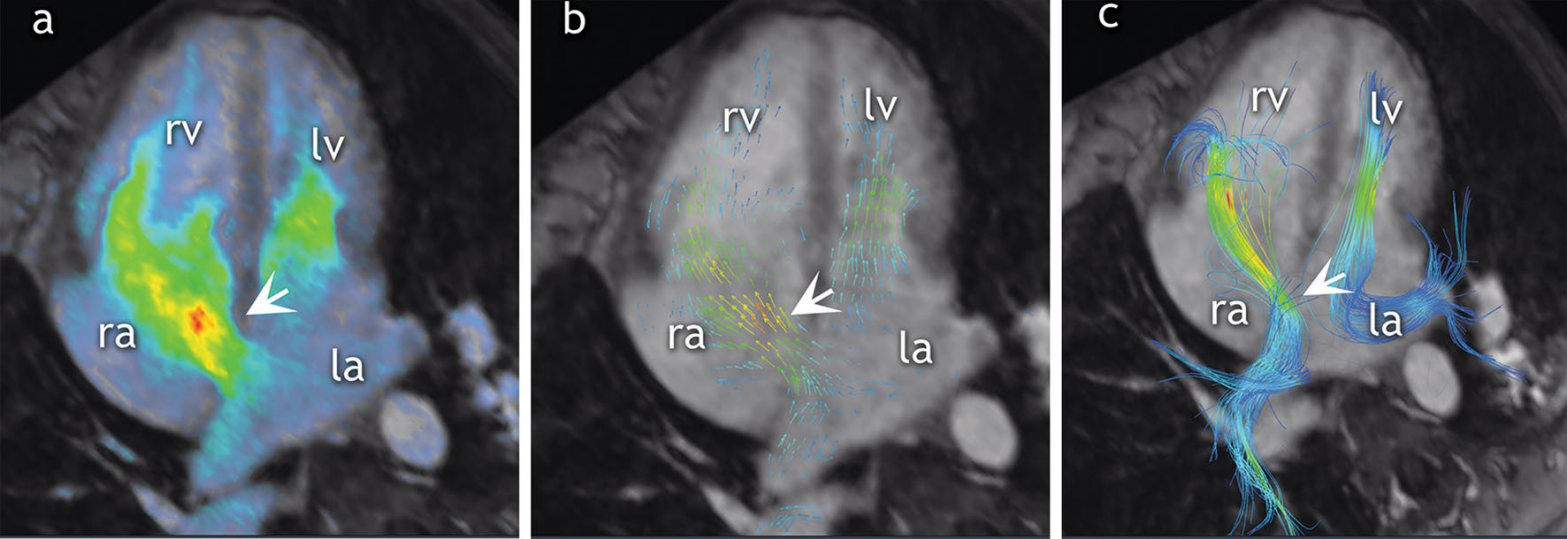
Multiple rendering techniques for ASD visualization. Color velocity overlay helps to identify the shunt in a frame-by-frame approach, but does not show flow directionality **a**. Flow direction is emphasized with vector overlay **b**. Streamlines help to track the flow that comes from pulmonary veins to the left atrium. In case of an atrial septum defect, the blood will cross to the right atrium and right ventricle. *Ra* right atrium, *rv* right ventricle, *la* left atrium, *lv* left ventricle, *SVC* superior vena cava

To detect and visualize the ASDs, volumetric data sets were reformatted in multiple orientations using several rendering techniques: color-coded velocity overlay, vector-velocity overlay, and streamlines (Fig. 1). ASDs were classified according to international guidelines [2, 5]. To visualize primum and secundum ASDs, the atrial septum was examined in short and long axis (Supplementary Fig. 1). Furthermore, to evaluate the presence of sinus venosus ASD, the superior and inferior cavo-atrial junctions were visualized. The coronary sinus was carefully assessed for detection of unroofed coronary sinus (Supplementary Fig. 2). In addition, streamlines were created from regions of interest in the pulmonary veins to further emphasize the flow across the atrial septum (Fig. 1). Incidental findings including the presence of a bicuspid aortic valve (BAV) and PAPVR were also documented.

**Fig. 2:**
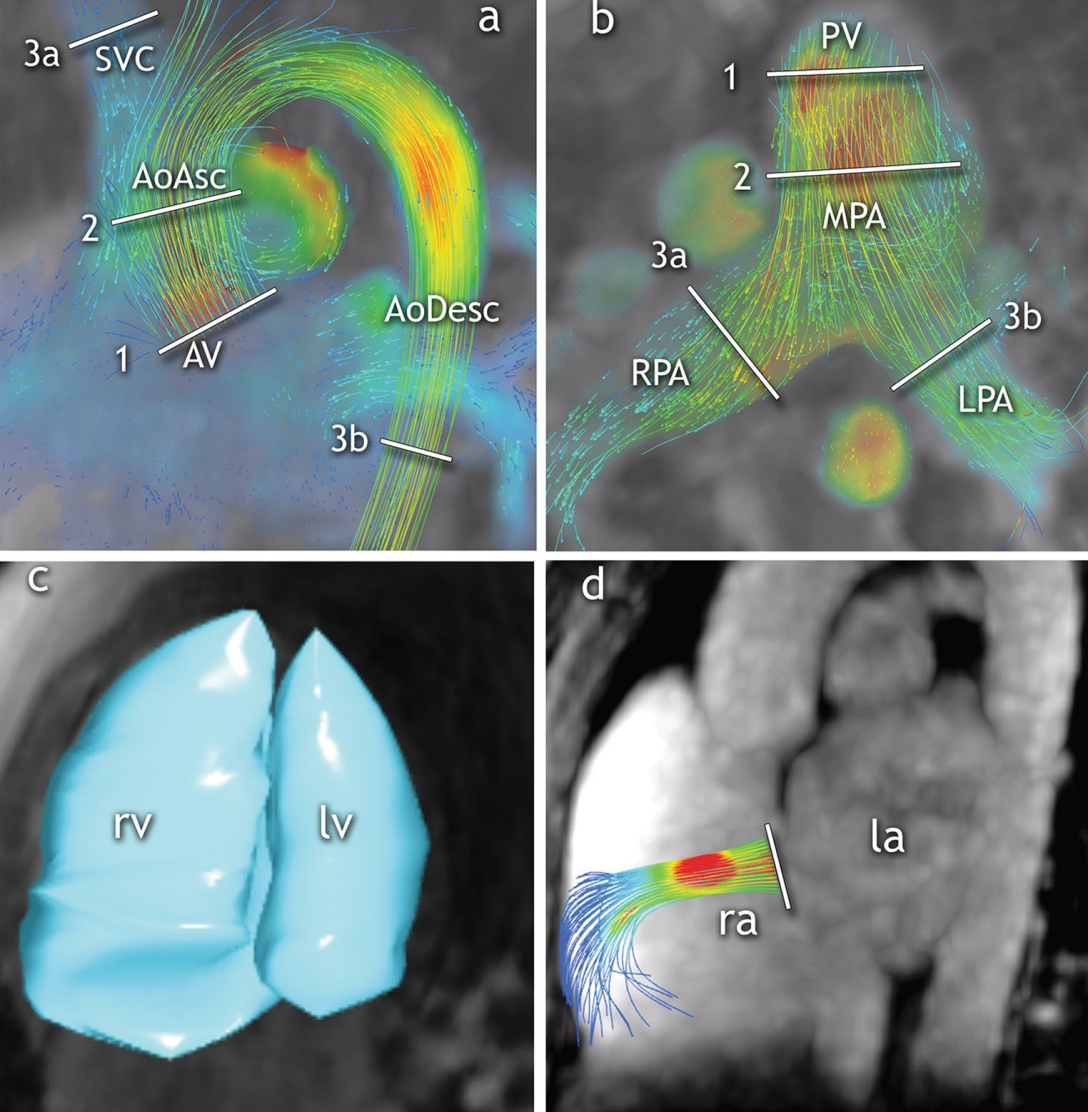
Levels of flow and shunt measurement. Systemic **a** and pulmonic **b** blood flows were measured at three different levels. Systemic flow **a** was measured at the aortic valve level [1], ascending aorta level [2] and as sum (3 = 3*a* + 3*b*) of the flow of the superior vena cava above the azygos vein (3*a*) and descending aorta (3*b*). When present, left persistent superior vena cava flow was added to the sum of SVC and descending aorta. Pulmonic flow **b** was measured at the pulmonary valve level [1], main pulmonary artery level [2] and as the sum of the right (3a) and left pulmonary artery flow (3b). Ventricular stroke volumes were calculated using magnitude images **c**. Additionally, shunt volumes were measured at the ASD level by septal tracking **d**

#### Quantification of flow and of shunt

Management of ASD is mostly driven by the severity of cardiac shunting, defined by the pulmonary (*Q*_p_) and systemic (*Q*_s_) blood flow ratio [2, 5]. Three levels were used to obtain *Q*_p_ and Q_s_ flow: (1) valve, (2) main artery and (3) periphery (Fig. 2). The valve and vessels were tracked and “contoured” throughout the entire cardiac cycle [19]. Shunt fractions (*Q*_p_/*Q*_s_) were calculated for each level in all patients.

Alternatively, ventricular stroke volumes may be used for shunt fraction calculation, provided that no significant (≤ 20%) valve insufficiency is present. The study population was screened for valvular insufficiency, which was quantified if detected. In the subset of patients with no significant valvular insufficiency, the shunt fraction was calculated additionally using the right ventricular (RVSV) and left ventricular (LVSV) stroke volume ratio. For this, end-diastolic and end-systolic ventricular volumes were segmented from the 4D flow magnitude anatomic images [20]. Lastly, shunt volumes were measured at the atrial level by tracking the atrial septum (Fig. 2). Indirect shunt volume quantification was obtained by subtracting systemic from pulmonary blood flow (*Q*_p_–*Q*_s_).

### Statistics

Statistical analysis was performed with SPSS software version 21 (IBM, New York, USA) and GraphPad Prism 4 Project (San Diego, CA, USA). Categorical variables are presented as number and percentages and continuous variables as mean (± standard deviation) or median (minimum-maximum). Correlation between measurements at different levels was evaluated using Spearman’s (rho) coefficient for nonparametric data, and agreement was analyzed with Bland-Altman plots [21]. The Spearman rho coefficient was classified as “very weak” for values of 0.00–0.19, “weak” for 0.20–0.39, “moderate” for 0.40–0.59, “strong” for 0.60–0.79 and “very strong” for 0.80–1.0 [22]. Interobserver reliability was assessed by intraclass correlation coefficient (ICC).

## Results

### Study population

Four-dimensional flow MRI examinations from 30 patients with ASD were gathered from the four academic centers. One nondiagnostic examination was excluded. (Supplementary Fig. 3). Mean age was 43 (± 17) years, and 11 (38%) patients were male. Six patients had two ASDs, and one patient had four ASDs. Of the 38 ASDs, 26 (68%) were classified as secundum, 4 (11%) primum and 8 (21%) sinus venosus [6 at the SVC level, 1 at the inferior vena cava (IVC) level and 1 unroofed coronary sinus]. Additional findings included PAPVR (*n* = 2) and BAV (*n* = 5).

### Correlation of 4D blood flow and stroke volume measurements

**Fig. 3:**
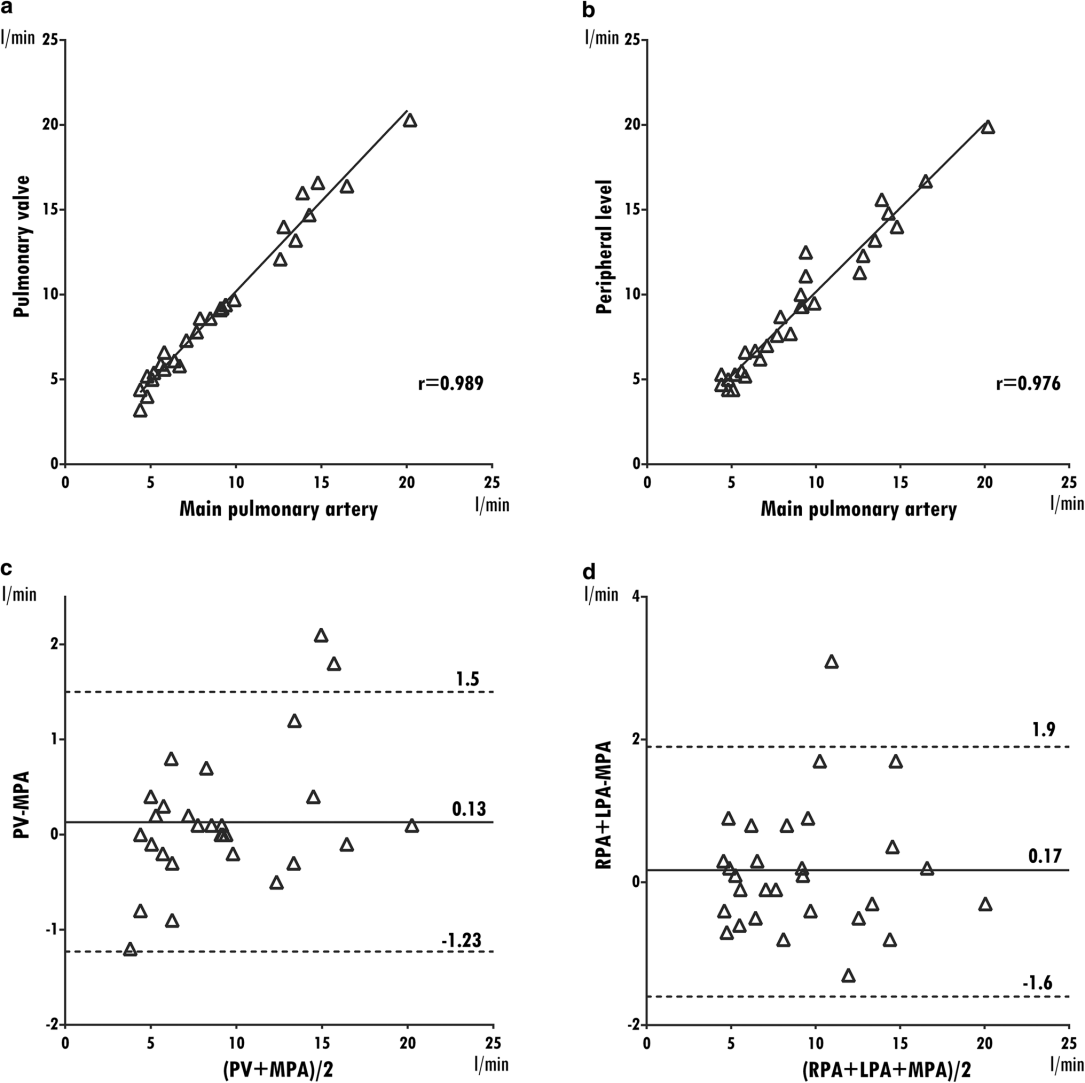
Pulmonary flow. Correlation and Bland-Altman plots of pulmonic flow measured at different levels using as reference the level of the main pulmonary artery. For each Bland-Altman plot, the average of measurements from both levels is plotted on the x-axis and the difference is plotted on the y-axis. The solid gray horizontal line plots the mean difference, and the dotted gray lines indicate the limits of agreement. *PV* pulmonary valve, *MPA* main pulmonary artery, *RPA* right pulmonary artery, *LPA* left pulmonary artery

Median Q_p_ was 8.5 l/min (4.4–20.2 l/min) at the main pulmonary artery level; 8.6 l/min (3.2–20.3 l/min) at the pulmonary valve level and 8.7 l/min (4.4–19.9 l/min) at the pulmonary branch level. Median cardiac output measured from RVSV was 9 l/min (4.6–20.0 l/min). The correlations between *Q*_p_ measurements performed at different levels were classified as very strong (Spearman’s rho = 0.885–0.886) (Fig. 3). Pulmonary flow measured in the main pulmonary artery also correlated well with right ventricular stroke volume (Spearman’s rho = 0.972). Relative to the main pulmonary artery, measurements at pulmonary valve and pulmonary branches overestimated flow by 0.15 l/min and 0.27 l/min, respectively (Fig. 3), while RVSV overestimated with 0.26 l/min. Bland-Altman, ranges, and biases are presented in Table 2.

**Table 2:** Consistency of flow measurements at different locations

Level of measurement	Flow (l/min) median (min–max)	Spearman’s rho^a^	Bland-Altman	
			Bias	± 1.96 SD
Pulmonic flow				
Pulmonary valve	8.6 (3.2–20.3)	0.886	0.148	− 0.86 to 1.16
Main pulmonary artery	8.5 (4.4–20.2)	–	–	–
Right + left pulmonary arteries	8.7 (4.4–19.9)	0.885	0.266	− 1.12 to 1.65
Right ventricular stroke volume^b^	9.0 (4.6–20.0)	0.972	− 0.257	− 2.01 to 1.49
Systemic flow				
Aortic valve	5.2 (2.9–10.0)	0.991	0.135	− 1.23 to 1.5
Ascending aorta	4.9 (2.7–10.7)	–	–	–
SVC^c^ + descending aorta	4.7 (2.9–10.5)	0.974	0.169	− 1.6 to 1.94
Left ventricular stroke volume^b^	4.8 (3.0–8.2)	0.906	0.157	− 2.45 to 2.1

Absolute blood flow measurements are consistent across multiple locations and with stroke volumes

*SVC* superior vena cava

^a^Main pulmonary artery and ascending aorta are taken as reference

^b^Eight patients had more than 20% mitral or tricuspid regurgitation and were excluded from the analysis

^c^SVC measured above the azygos vein when visible

**Fig. 4:**
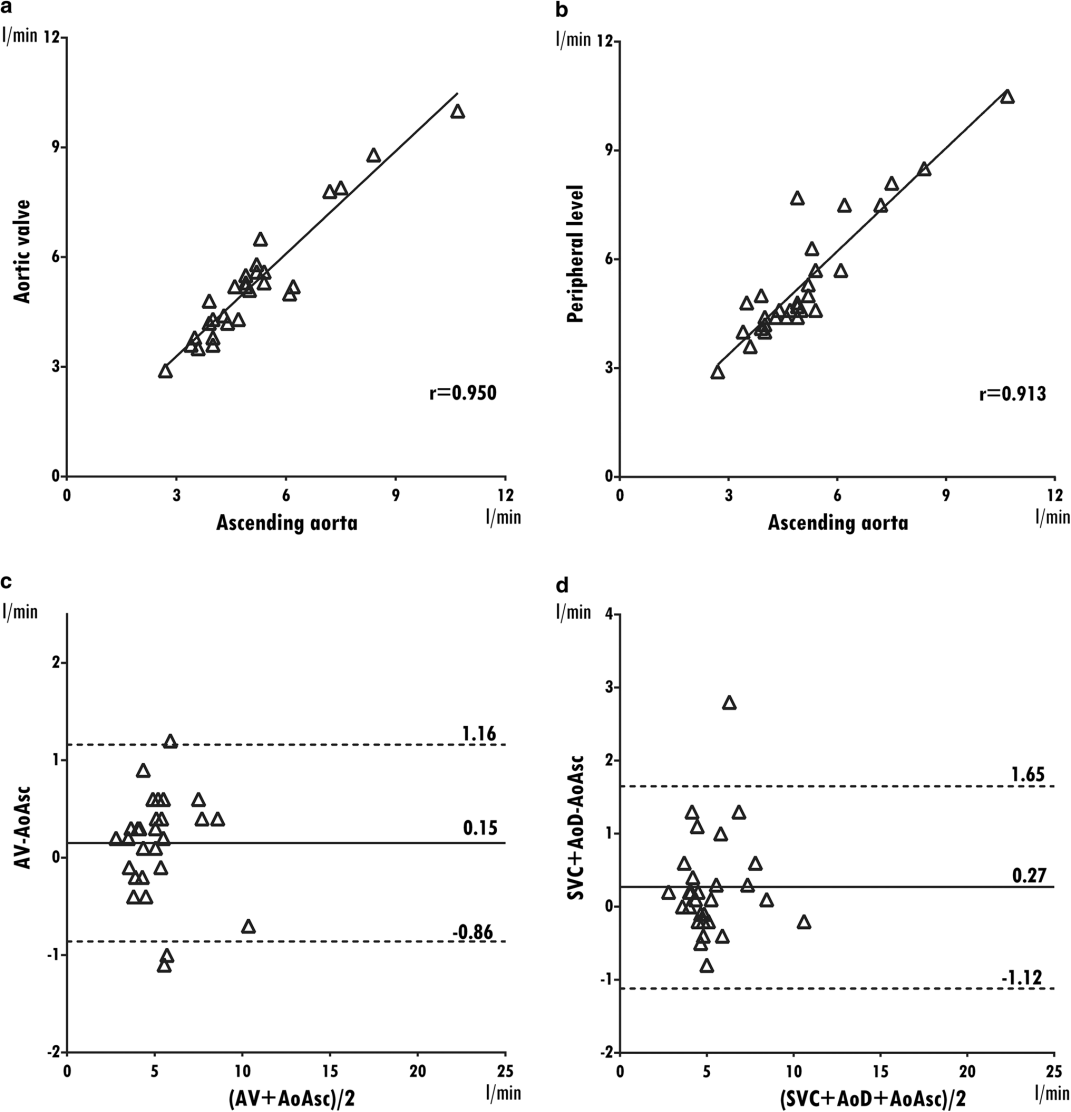
Systemic flow. Correlation and Bland-Altman plots of systemic flow measurements at different levels and taking as reference the level of the ascending aorta. For each Bland-Altman plot, the average of measurements from both levels is plotted on the x-axis and the difference is plotted on the y-axis. The solid gray horizontal line plots the mean difference, and the dotted gray lines indicate the limits of agreement. *AV* aortic valve, *AoAsc* ascending aorta, *AoD* descending aorta, *SVC* superior vena cava

For systemic flow, median *Q*_s_ is 4.9 l/min (2.7–10.7 l/min) at the ascending aorta level, 5.2 l/min (2.9–10.0 l/min) at the aortic valve level and 4.7 l/min (2.9–10.5 l/min) at the peripheral level. Median cardiac output measured from LVSV was 4.8 l/min (3.0–8.2 l/min). Similarly, the correlations between *Q*_s_ measurements performed at different levels were classified as very strong (Spearman’s rho = 0.991–0.974) (Table 2, Fig. 4). Relative to the ascending aorta results, measurements at the aortic valve overestimated *Q*_s_ with 0.14 l/min, and by 0.17 l/min at the peripheral level, while measurements of the LVSV underestimated *Q*_s_ with 0.16 l/min (Fig. 4). Bland-Altman, ranges, and biases are presented in Table 2.

### Correlation of 4D flow shunt fraction measurements

**Fig. 5:**
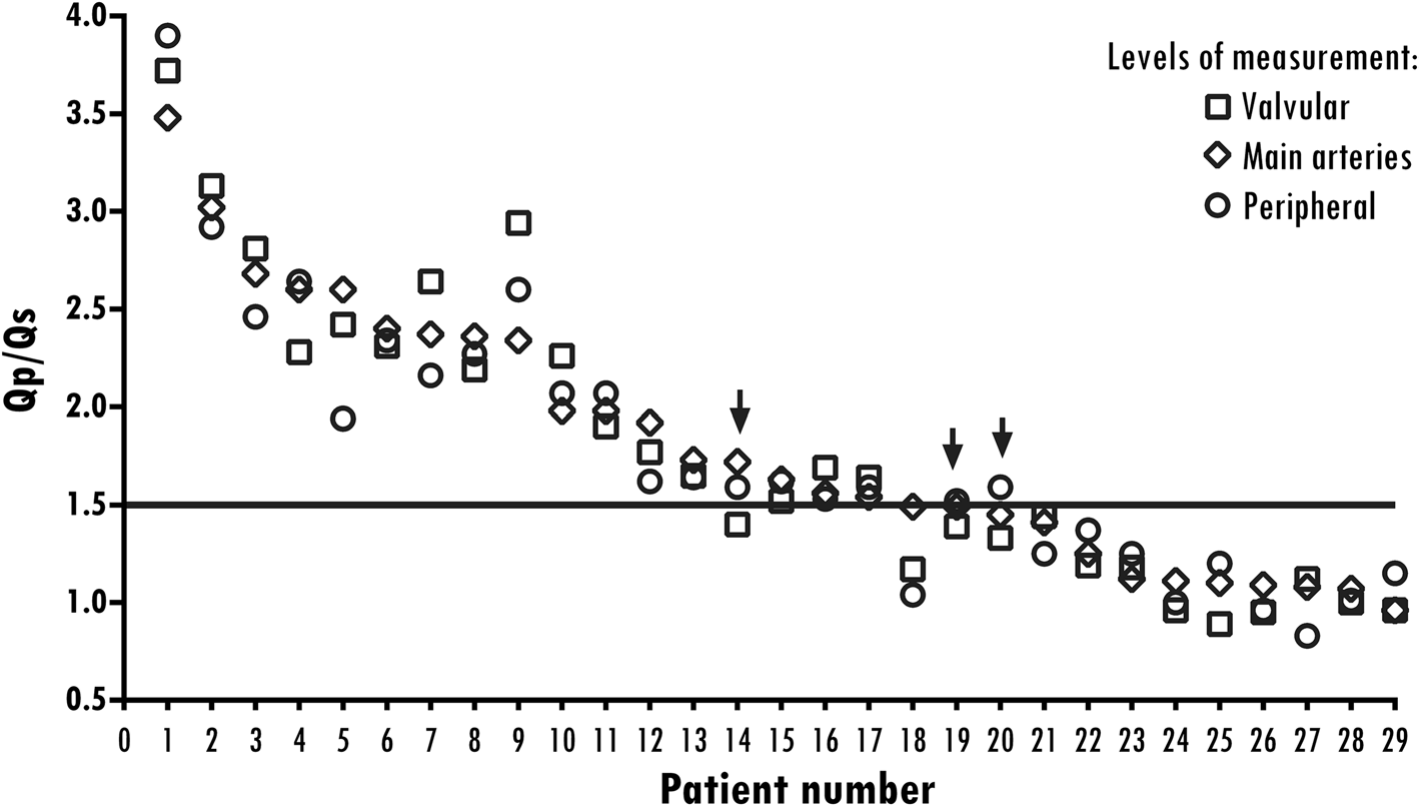
Distribution of atrial septal defect shunt fractions (*Q*_p_/*Q*_s_) measured at multiple levels. *Q*_p_/*Q*_s_ at different locations ordered from high to low. A solid line is placed at the treatment threshold of 1.5 *Q*_p_/*Q*_s_. Arrows highlight the three patients in which the measurements at different levels are crossing the threshold line

Good correlation was found between *Q*_p_/*Q*_s_ ratios derived at the main artery level and ratios derived at the valve and peripheral level (Spearman’s *ρ* = 0.95 for both comparisons), with smaller bias for the valve level vs. peripheral level (− 0.023 l/min vs. − 0.049 l/min, *p* = 0.922) (Supplementary Fig. 4). Bland-Altman, ranges, and biases are presented in Table 3. A threshold of 1.5 is often used as a threshold for ASD closure. In our cohort, 10 patients had ratios measuring below 1.5 at all levels, and 16 patients had shunt ratios measuring above 1.5 at all levels. In only three patients did we observe some measurements above and below the 1.5 threshold, and at all locations these measurements tended to stay close to 1.5 (range 1.3–1.7) (Fig. 5).

**Table 3:** Comparison of shunt fractions measured at different locations

Level of measurement	Shunt fraction median (min–max)	Spearman’s rho^a^	Bland-Altman	
			Bias	± 1.96 SD
Valve	1.6 (0.9–3.7)	0.95	− 0.023	− 0.42 to 0.37
Main artery^a^	1.6 (1.0–3.5)			
Peripheral	1.6 (0.8–3.9)	0.95	− 0.049	− 0.47 to 0.37
Stroke volume^b^	1.8 (1.0–3.3)	0.93	0.072	− 0.57 to 0.71

^a^Level of main arteries is taken as reference

^b^Eight patients had more than 20% mitral or tricuspid regurgitation and were excluded from the analysis

### Interobserver consistency of 4D flow, shunt fraction and volume measurements

Measurements of blood flow, shunt fraction and shunt volume with 4D flow were highly reproducible between two independent readers. Interobserver consistency for flow measurements was excellent at all levels, showing ICCs all ≥ 0.955. Interobserver consistency for the calculated shunt fraction showed ICCs ≥ 0.98 and for shunt volume ICCs ≥ 0.979. All ICCs for measurements of blood flow, calculated shunt fractions (*Q*_p_/*Q*_s_) and shunt volumes are displayed in Table 4.

**Table 4:** Comparison of flow measurements by independent observers

Level	Systemic flow	Pulmonary flow	*Q*_p_/*Q*_s_	Shunt volume
Valve	0.968	0.987	0.983	0.989
Main artery	0.975	0.986	0.981	0.987
Peripheral	0.955	0.981	0.968	0.979

Intraclass coefficients (ICCs) for measurements of blood flow, calculated shunt fractions (*Q*_p_/*Q*_s_) and shunt volumes displayed

### Direct versus indirect 4D shunt volume measurements

In 20/29 patients (69%), it was feasible to obtain direct shunt volumes at the exact location of the septal defect. Correlations between direct and indirect (*Q*_p_–*Q*_s_) measurements were classified as very strong (Spearman’s *ρ* = 0.96). However, shunt volume was underestimated by 0.57 l/min using direct measurements. Bland-Altman, ranges and standard deviation are presented in Table 5. Two of these patients had multiple ASDs, but some small ASDs did not allow for direct flow measurement (Supplementary Fig. 5).

**Table 5:** Comparison of shunt volume measured at the atrial septal level against measurement at the main artery level

Level of measurement	Shunt volume (l/min) median (min–max)	Spearman’s rho^†^	Bland-Altman	
			Bias (l/min)	± 1.96 SD
Atrial septum^∞^	2.95 (0.2–8.7)	0.955	− 0.57	− 2.71 to 1.57
Main artery^†^	3.6 ( − 0.2 to 1.8)	−	–	–

^∞^Direct measurement of ASD was feasible in 20 patients

^†^Level of main arteries is taken as reference

## Discussion

We show in this study that 4D flow MRI can be sufficient for evaluation of patients with ASD, including quantification of shunt fraction, and can be robustly performed at multiple institutions; 4D flow MRI is consistent and reliable for measuring systemic and pulmonic blood flow and obtaining shunt fractions at multiple levels across the vascular tree. In daily practice, a shunt fraction (*Q*_p_/*Q*_s_) threshold above 1.5 is often used as a critical parameter to determine the need for ASD closure. By 4D flow MRI, few patients had mixed results near the 1.5 threshold. In those patients, other clinical features may be used to decide upon individual surgical or medical management, such as right heart chamber enlargement or pulmonary pressure [2, 5].

In this study, direct shunt volume quantification was obtained at the level of ASD by tracking the atrial septum frame by frame throughout the cardiac cycle. Direct shunt volume quantification was feasible in 69% of the patients and correlated well with calculated shunt volumes obtained by 4D flow measurements at the level of main arteries (*Q*_p_–*Q*_s_) (*r* = 0.955). Tracking the atrial septum may be challenging if there is insufficient image quality, if the size of the ASD is small or if there are multiple ASDs. For example, in two patients the direct quantification value was lower than the indirect quantification. When these cases were further reviewed, we found additional shunts, which were missed in the initial analysis (Supplementary Fig. 5). Therefore, we believe that direct quantification of each ASD can be helpful to determine whether all of shunts have been appropriately accounted for. Mismatch between direct and indirect measurements may point to additional undetected shunts.

We show in the current study that it is possible to achieve excellent multilevel and interreader reproducibility with 4D flow MRI at multiple centers. This alleviates some previous concerns that 4D flow might only be achievable at one or two centers with extensive experience. This is further supported by recent studies showing good scan-rescan reproducibility and good intraobserver agreement with 4D flow [23, 24]. We further show here that experienced readers are not necessarily required to achieve high reproducibility. In addition, we demonstrate here that 4D flow can enable measurement of shunts at multiple alternative locations. This is especially helpful in the case of turbulent flow, aliasing or metallic artifacts. The 4D flow measurements can be performed at an alternative location distant to such artefacts to answer the clinical question. In patients with BAV, for example, flow acceleration across the aortic valve [25] can compromise the accuracy of measurements in areas of turbulent flow [26, 27], and an alternate measurement may be more accurate.

We present the current work, recognizing that 4D flow is an evolving imaging technique [28, 29] and new strategies are being developed, including incorporation of multiple velocity encoding speeds [30, 31]. To date, 4D flow has shown its potential for evaluation of congenital heart disease [32], and is being introduced in daily clinical practice for other clinical indications [33]. Additional work may be required to assess the performance of 4D flow in specific clinical scenarios. In the current work, we did not explore a direct comparison to other advanced imaging techniques, which can also be used to assess shunt fraction. For example, Yamasaki et al. propose CT as an approach for quantification of ASD [34], obtaining ventricular stroke volumes from finely detailed anatomic data. However, without flow information provided by 4D flow or echocardiography, this approach may be confounded by concomitant valve regurgitation.

### Limitations

We recognize a few potential limitations of the study. The patient population was not large, but we believe sufficient to demonstrate the robustness of the method. Second, although it is a study across multiple centers, all 4D flow acquisitions were performed using equipment from a single vendor. Additional work may be required to confirm similar quality 4D flow measurements can be obtained on other platforms. Third, a gadolinium-based contrast agent was used prior to image acquisition at all sites. Further work is needed to determine whether similar results can be obtained without intravenous contrast. In addition, we did not perform a direct comparison against 2D phase-contrast MRI in this study. We did find that flow measurements were consistent with stroke volumes obtained from anatomical data, which was reassuring. Multiple previous studies have compared 4D flow and 2D phase-contrast measurements, showing that measurements from each technique are generally consistent [35, 36]. Bollache and colleagues showed better correlation between 4D flow and three-direction-velocity 2D phase-contrast than with the one-direction-velocity 2D phase-contrast technique, which is the most commonly used clinical technique [36].

## Conclusions

For patients referred for evaluation of ASD, 4D flow MRI showed excellent multilevel and interreader reproducibility for systemic and pulmonary blood flow measurements and shunt quantification obtained at different levels throughout the vascular tree.

## Electronic supplementary material

Below is the link to the electronic supplementary material.

**Figure 1** Supplementary. Reconstruction planes. Long- **a** and short-axis **b** views of the atrial cavities were used to evaluate the presence of ASD. *Ra* right atria, *rv* right ventricle, *la* left atria, *lv* left ventricle, *SVC* superior vena cava (JPEG 1549 kb)

**Figure 2** Supplementary. Classification of ASD subtypes. ASD secundum **a** can easily be identified as it is centered in the atrial septum. ASD primum **b** is typically located near the ventricles as it is a type of atrioventricular defect, and the associated mitral cleft can lead to mitral regurgitation. Sinus venous ASD **c** is located more cranially at the connection of SVC and RA. Unroofed coronary sinus **d** can be detected as a connection between the LA and coronary sinus. *Ra* right atrium, *rv* right ventricle, *la* left atrium, *lv* left ventricle, *SVC* superior vena cava (JPEG 2982 kb)

**Figure 3** Supplementary. Chart of patient inclusion and measurements performed (JPEG 1633 kb)

**Figure 4** Supplementary. Shunt fractions (*Q*_p_/*Q*_s_) measured at different levels. Correlations and Bland-Altman plots of the *Q*_p_/*Q*_s_ fractions, taking as reference the *Q*_p_/*Q*_s_ at the level of main arteries. For each Bland-Altman plot, the average of fractions from both levels is plotted on the x-axis and the difference is plotted on the y-axis. The solid gray horizontal line plots the mean difference, and the dotted gray lines indicate the limits of agreement (JPEG 2982 kb)

**Figure 5** Supplementary. Example of a patient with multiple ASDs. A 48-year-old female patient with four ASDs as identified using 4D flow MRI (presented short axes, **a**–**b**). One large and three small ASDs were detected (arrows). *La* left atrium, *ra* right atrium, 1–4 the ASD numbers (JPEG 1433 kb)
