# Supplementary figures and images for: Evaluation of atrial septal defects with 4D flow MRI—multilevel and inter-reader reproducibility for quantification of shunt severity
Source: MAGMA. 2018 Aug 31;32(2):269–79. doi: 10.1007/s10334-018-0702-z (PMC6424937; doi:10.1007/s10334-018-0702-z)

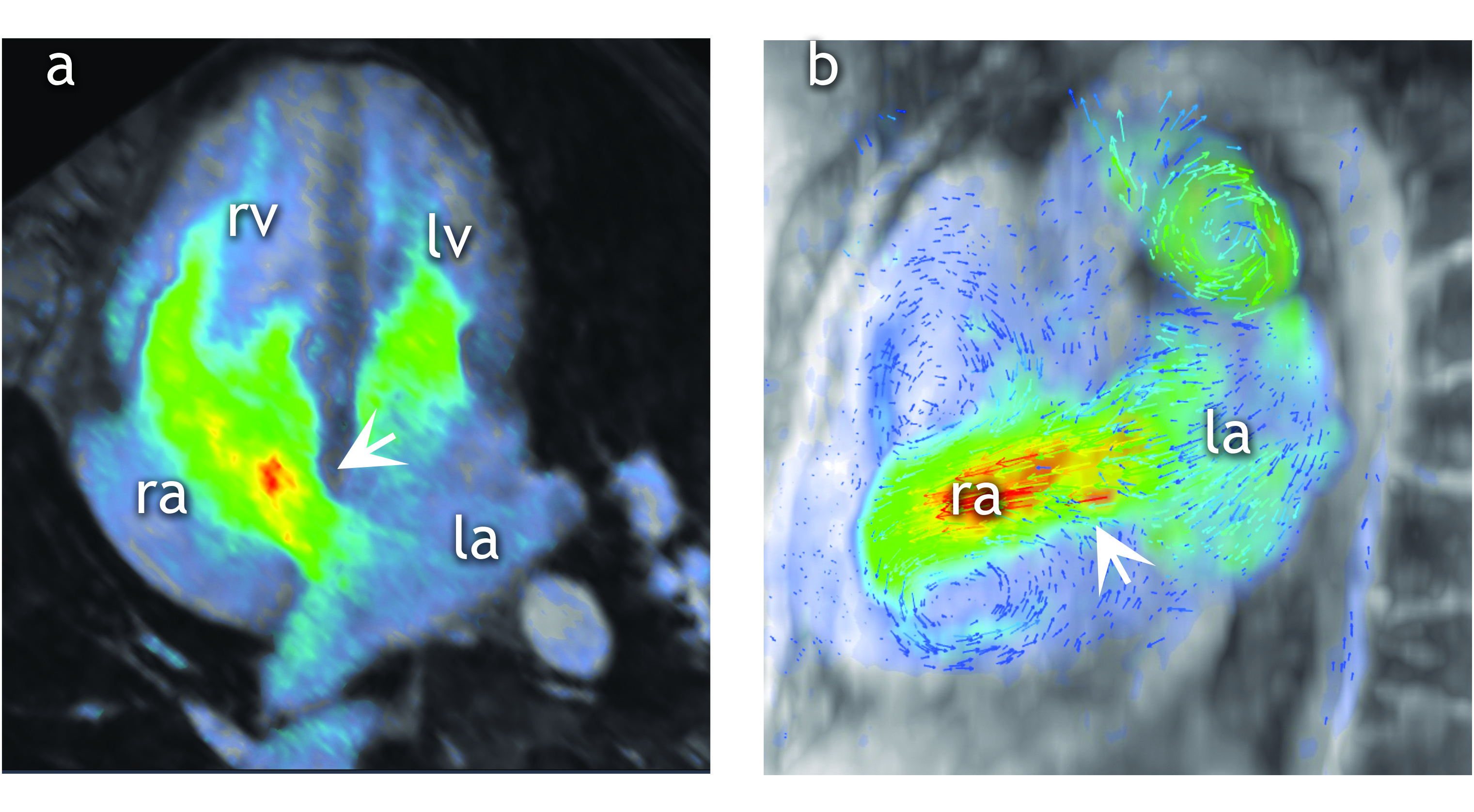

Supplement: Supplementary file 1 — Figure 1 Supplementary. Reconstruction planes. Long- a and short-axis b views of the atrial cavities were used to evaluate the presence of ASD. Ra right atria, rv right ventricle, la left atria, lv left ventricle, SVC superior vena cava (JPEG 1549 kb) [file 10334_2018_702_MOESM1_ESM.jpg]

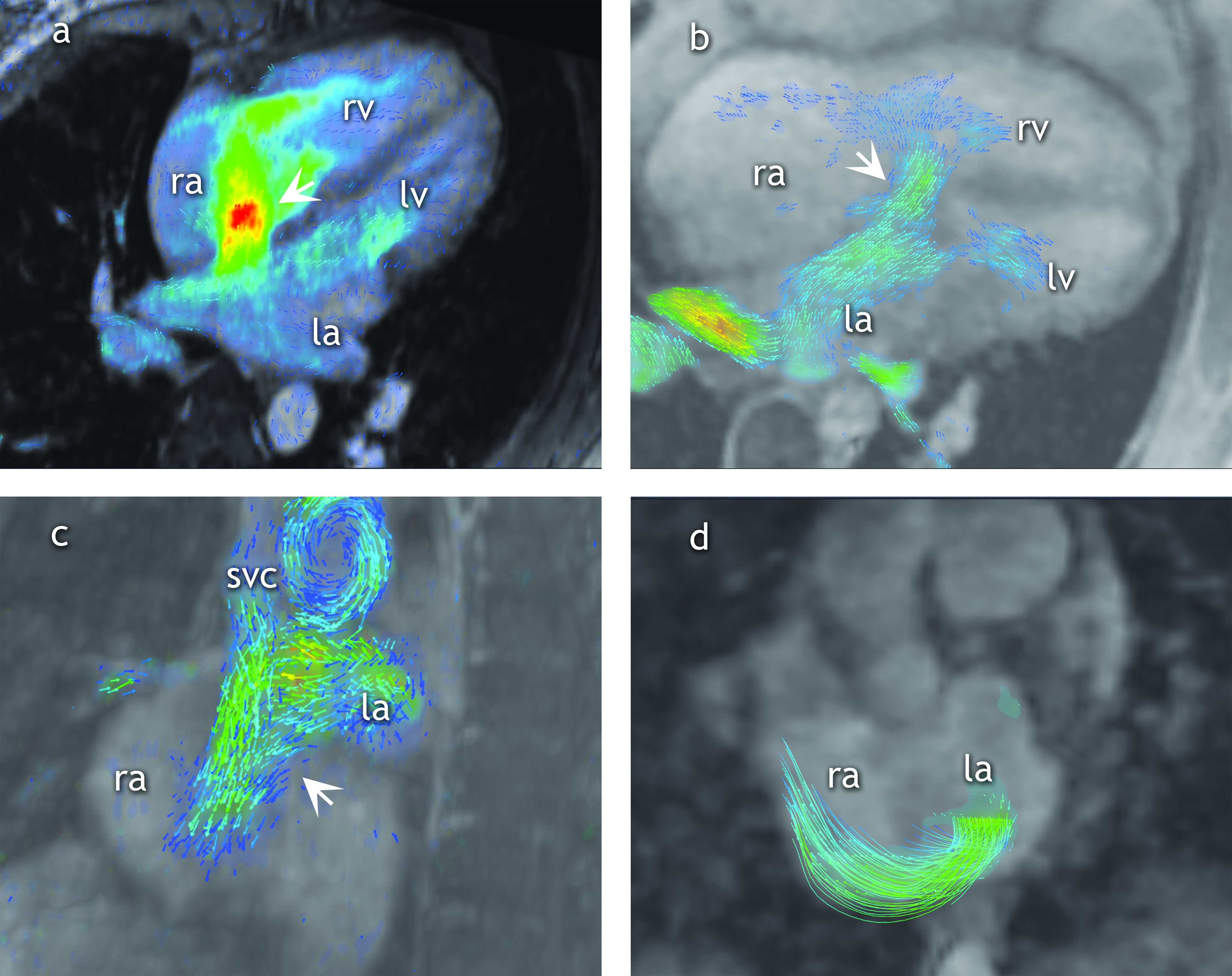

Supplement: Supplementary file 2 — Figure 2 Supplementary. Classification of ASD subtypes. ASD secundum a can easily be identified as it is centered in the atrial septum. ASD primum b is typically located near the ventricles as it is a type of atrioventricular defect, and the associated mitral cleft can lead to mitral regurgitation. Sinus venous ASD c is located more cranially at the connection of SVC and RA. Unroofed coronary sinus d can be detected as a connection between the LA and coronary sinus. Ra right atrium, rv right ventricle, la left atrium, lv left ventricle, SVC superior vena cava (JPEG 2982 kb) [file 10334_2018_702_MOESM2_ESM.jpg]

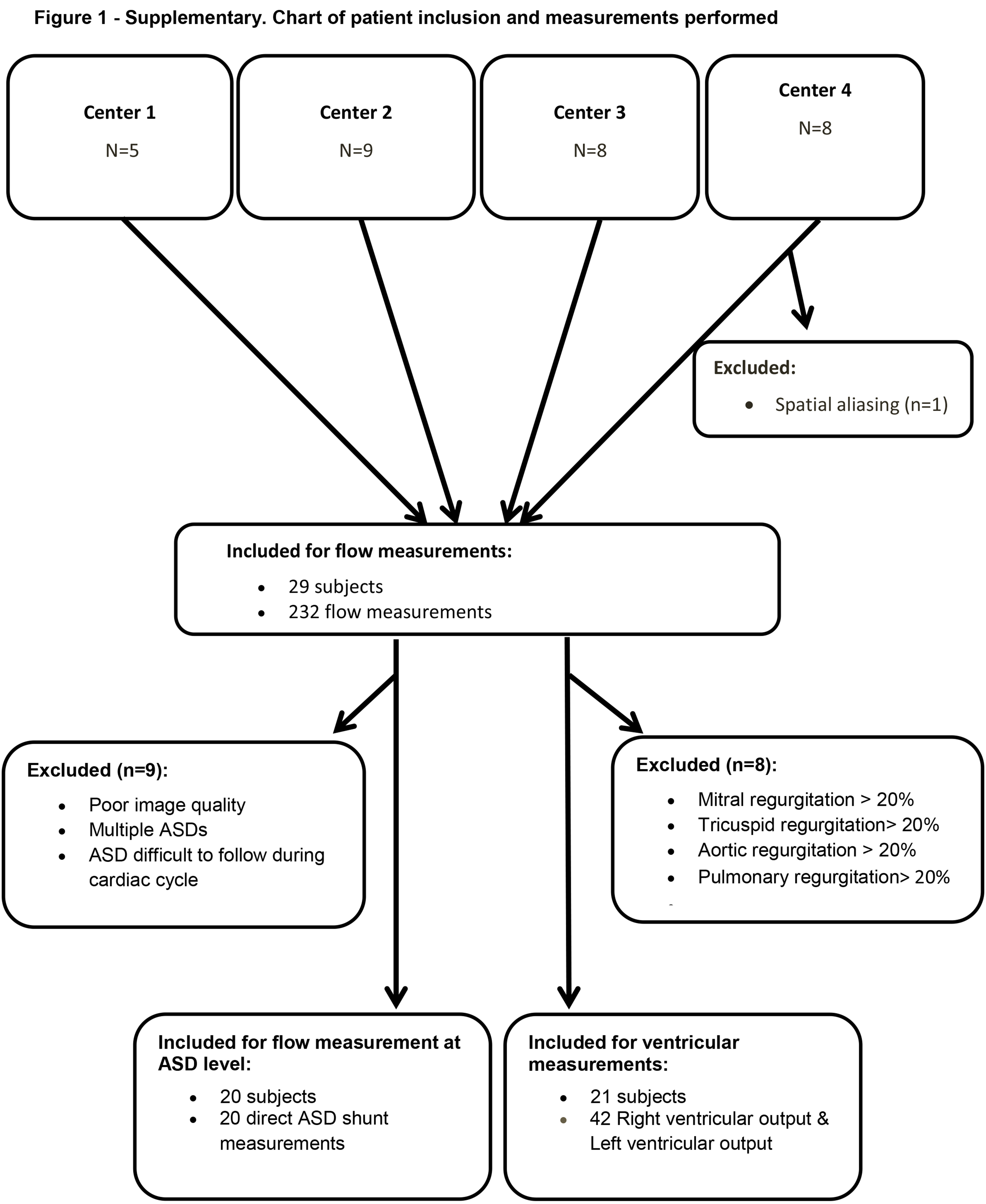

Supplement: Supplementary file 3 — Figure 3 Supplementary. Chart of patient inclusion and measurements performed (JPEG 1633 kb) [file 10334_2018_702_MOESM3_ESM.jpg]

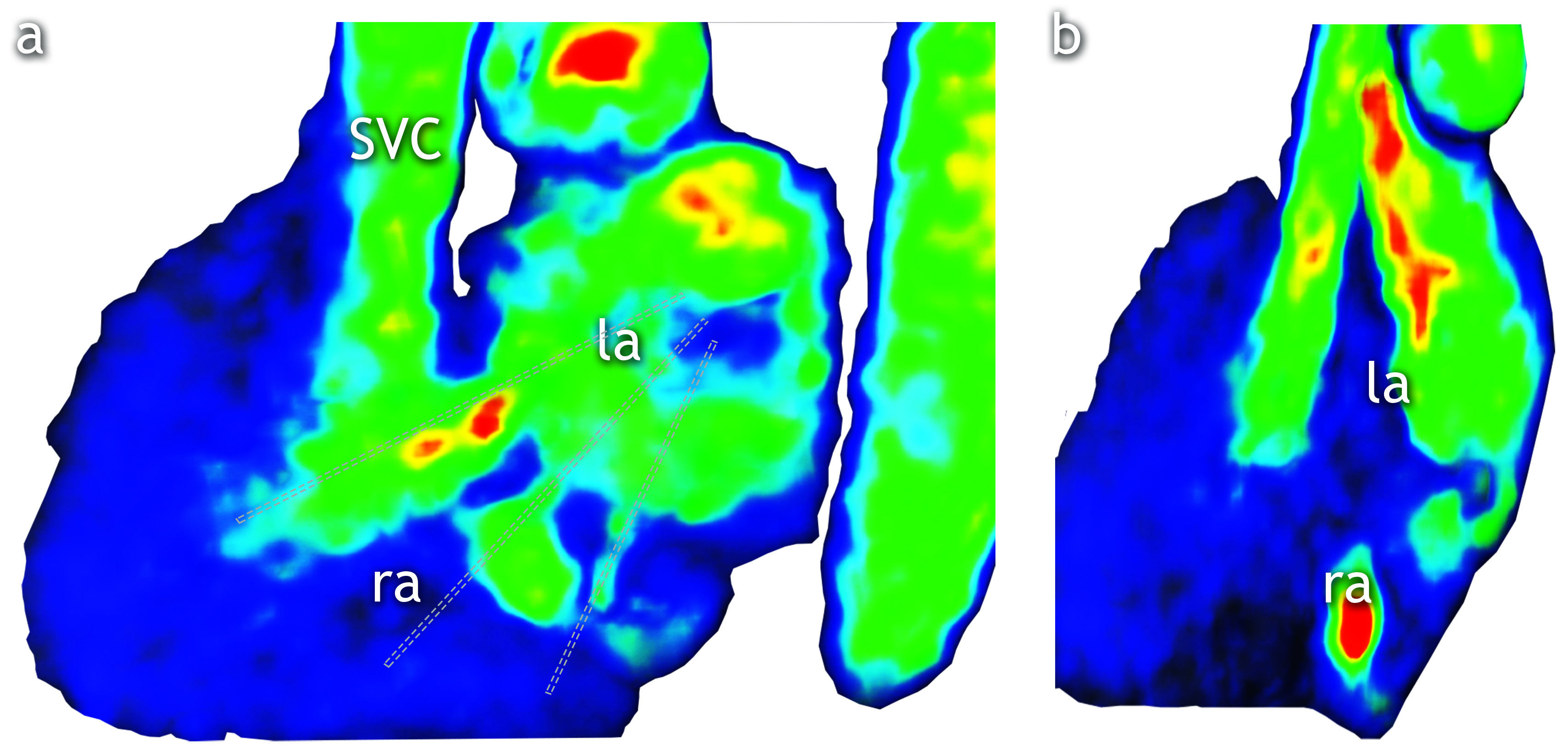

Supplement: Supplementary file 5 — Figure 5 Supplementary. Example of a patient with multiple ASDs. A 48-year-old female patient with four ASDs as identified using 4D flow MRI (presented short axes, a–b). One large and three small ASDs were detected (arrows). La left atrium, ra right atrium, 1–4 the ASD numbers (JPEG 1433 kb) [file 10334_2018_702_MOESM5_ESM.jpg]
